# Supplementary material for: Pregistered movie-fMRI analyses reveal altered visual feature encoding in autism in pSTS
Source: bioRxiv. 2026 Mar 24:2026.03.23.713749. Preprint. [Version 1] doi: 10.64898/2026.03.23.713749 (PMC13041991; doi:10.64898/2026.03.23.713749)
Supplement: 1 [file NIHPP2026.03.23.713749V1-supplement-1.pdf]

bioRxiv preprint doi: https://doi.org/10.64898/2026.03.23.713749; this version posted March 24, 2026. The copyright holder for this preprint (which was not certified by peer review) is the author/funder, who has granted bioRxiv a license to display the preprint in perpetuity. It is made available under aCC-BY 4.0 International license.

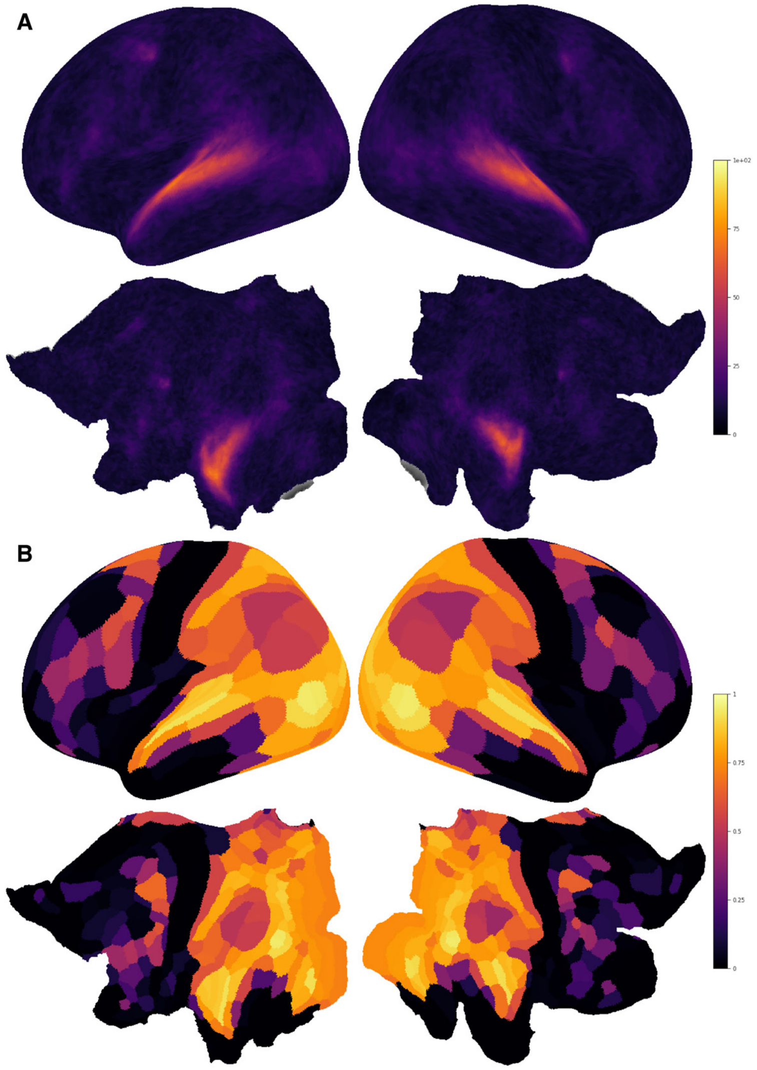

Figure 2—figure supplement 1. Overall model performance across the cortex. **(A)** Whole-brain plot of the percentage of subjects with a significant grayordinate at each region. The significance of each grayordinate was tested via a null model by repeatedly temporally permuting the order of observations and retraining and testing the models over 1,000 permutations. **(B)** The Spearman-Brown corrected split-half noise ceilings for each MMP parcel.

bioRxiv preprint doi: https://doi.org/10.64898/2026.03.23.713749; this version posted March 24, 2026. The copyright holder for this preprint (which was not certified by peer review) is the author/funder, who has granted bioRxiv a license to display the preprint in perpetuity. It is made available under aCC-BY 4.0 International license.

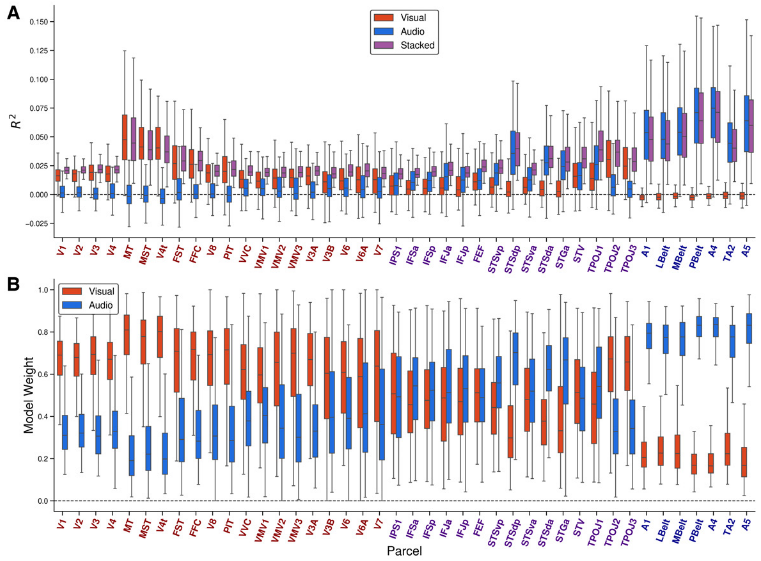

Figure 2—figure supplement 2. (A) Model performance (R^2) for the visual (red), audio (blue), and stacked (purple) encoding models across perceptual regions of interest for all included participants (both ASD and nonASD). **(B)** Visual (red) and audio (blue) model weights from the stacked encoding model.

bioRxiv preprint doi: https://doi.org/10.64898/2026.03.23.713749; this version posted March 24, 2026. The copyright holder for this preprint (which was not certified by peer review) is the author/funder, who has granted bioRxiv a license to display the preprint in perpetuity. It is made available under aCC-BY 4.0 International license.

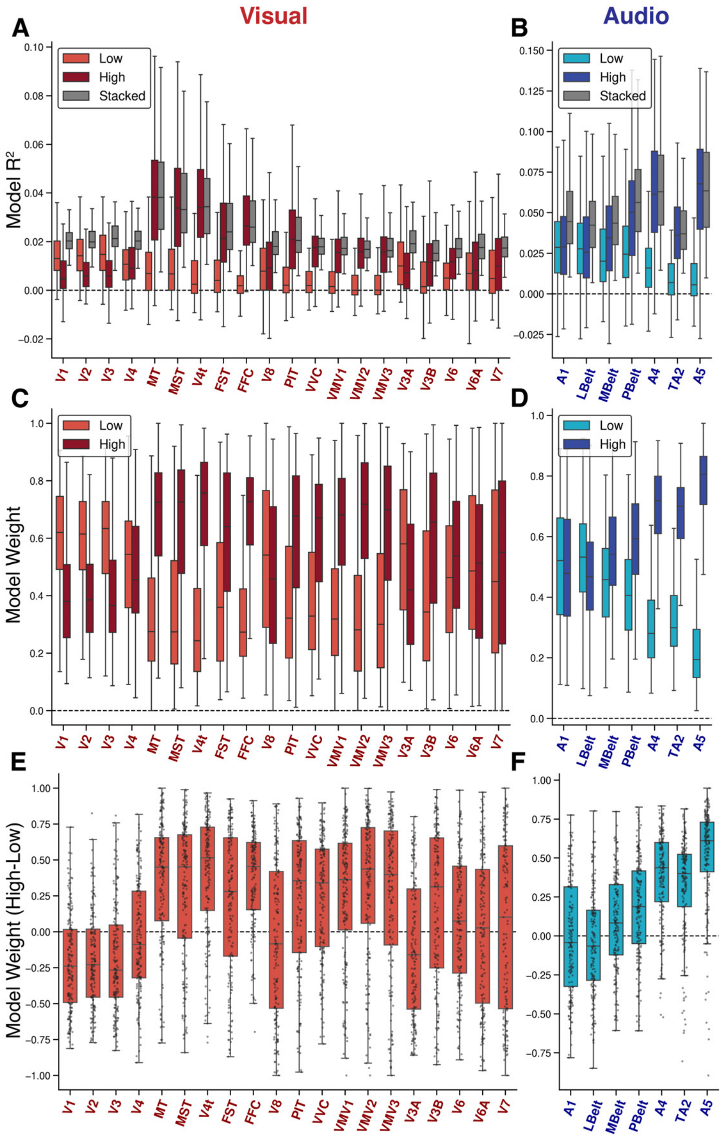

Figure 2—figure supplement 3. **(A)** Model performance (R2) for the visual low- (light red) and high-level (dark red) and stacked (grey) encoding models across all nonASD and ASD participants. **(A)** Corresponding R2 for audio models. **(C)** Model weights from the stacked visual encoding model. **(D)** Model weights from the stacked audio encoding model. **(E)**, **(F)** High- vs. low-level visual and audio perceptual preferences ($W_H - W_L$) are calculated by taking the difference of high- and low-level weights (shown in **B** and **C**). Perceptual preference is in the range of -1 to 1, as the stacked encoding model weights range from 0 to 1. Positive values here indicate a high-level preference, negative values indicate a low-level preference, and values around zero indicate no preference.

bioRxiv preprint doi: https://doi.org/10.64898/2026.03.23.713749; this version posted March 24, 2026. The copyright holder for this preprint (which was not certified by peer review) is the author/funder, who has granted bioRxiv a license to display the preprint in perpetuity. It is made available under aCC-BY 4.0 International license.

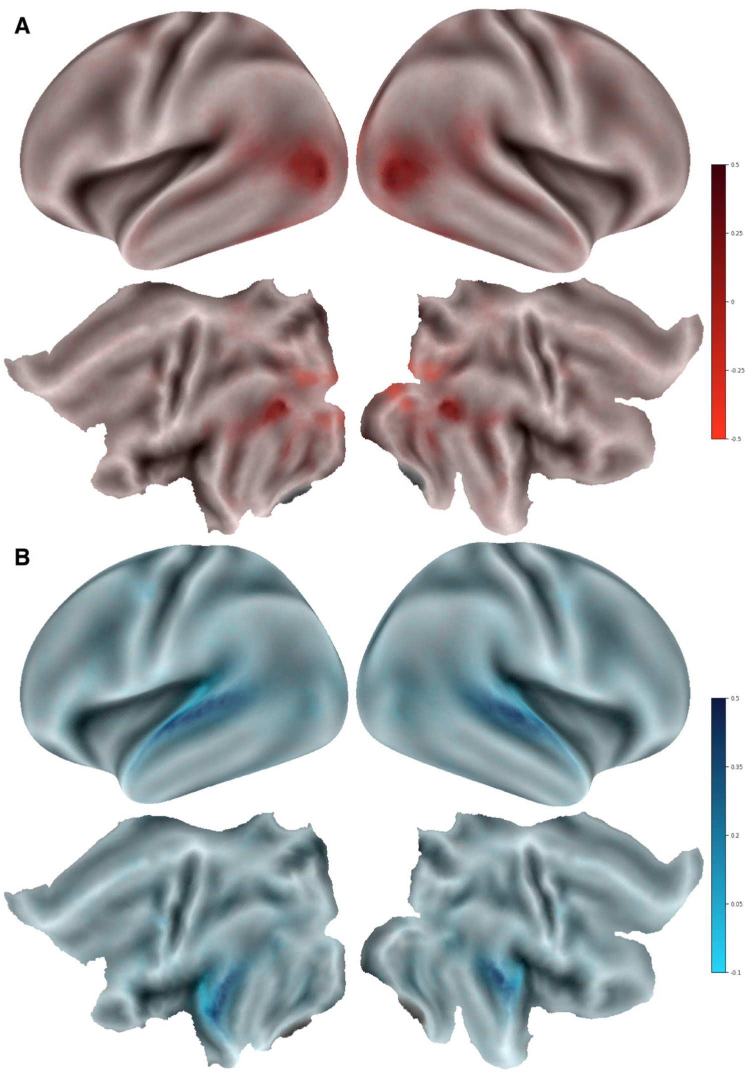

Figure 2—figure supplement 4. **(A)** Whole-brain grayordinate-wise plot of mean high- vs. low-level perceptual preference across all participants (ASD and nonASD). **(B)** The same corresponding plot but from the audio stacked encoding model.

bioRxiv preprint doi: https://doi.org/10.64898/2026.03.23.713749; this version posted March 24, 2026. The copyright holder for this preprint (which was not certified by peer review) is the author/funder, who has granted bioRxiv a license to display the preprint in perpetuity. It is made available under aCC-BY 4.0 International license.

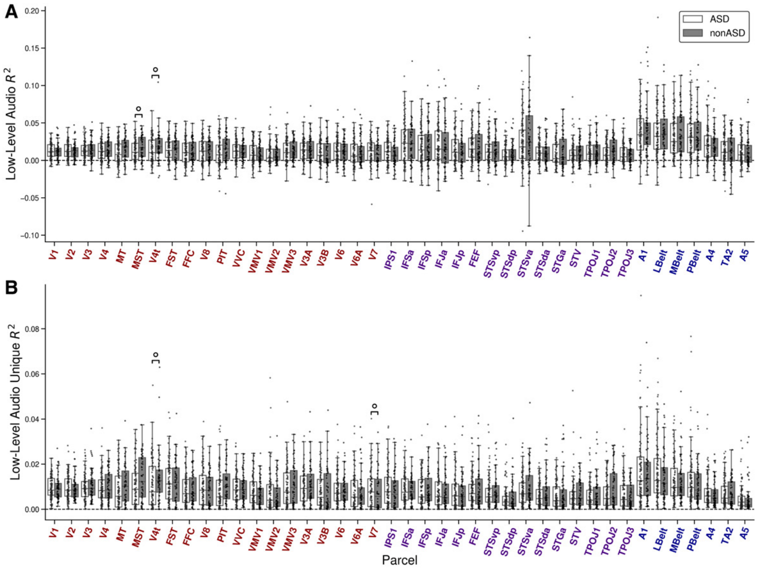

Figure 3—figure supplement 1. (A) Box plot of low-level audio encoding model explained variance (R^2) for ASD (white) and nonASD (gray) groups across all perceptual ROIs. (B) Box plot of the unique explained variance (R_u^2). The results correspond to the 40% FD threshold. Boxes annotated with circles indicate an initially significant difference between groups that did not survive FDR correction. Boxes show the quartiles of the dataset, and whiskers show the distribution, with the exception of outliers. Each dot is the mean R^2 from a single subject from all statistically significant grayordinates within each ROI.

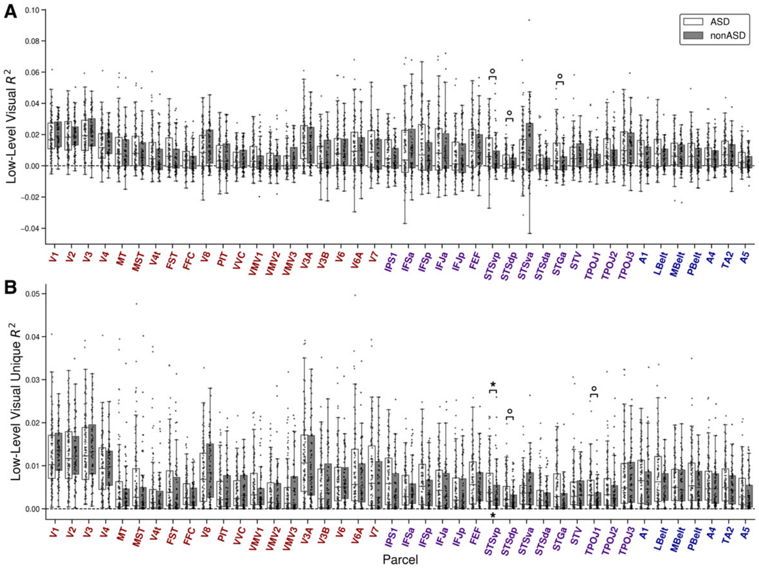

Figure 3—figure supplement 2. (A) Box plot of low-level visual encoding model R^2 for ASD (white) and nonASD (gray) groups across all perceptual ROIs. (B) Corresponding box plot of R_u^2. Results correspond to the 40% FD threshold. Boxes annotated with an asterisk indicate a significant group difference (FDR p<0.05), while a circle indicates an initially significant difference between groups that did not survive FDR correction. Boxes show the quartiles of the dataset and whiskers show the distribution with the exception of outliers. Each dot is the mean R^2 from a single subject from all statistically significant grayordinates within each ROI.

bioRxiv preprint doi: https://doi.org/10.64898/2026.03.23.713749; this version posted March 24, 2026. The copyright holder for this preprint (which was not certified by peer review) is the author/funder, who has granted bioRxiv a license to display the preprint in perpetuity. It is made available under aCC-BY 4.0 International license.

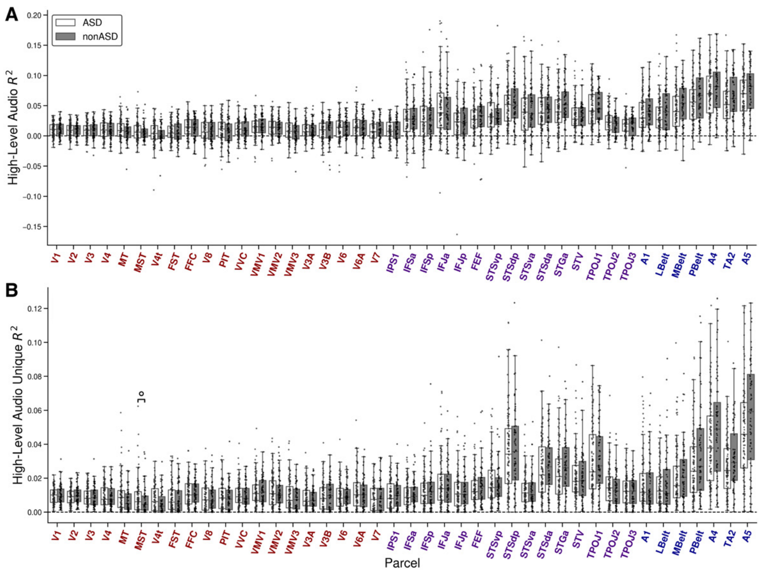

Figure 3—figure supplement 3. (A) Box plot of high-level audio encoding model R^2 for ASD (white) and nonASD (gray) groups across all perceptual ROIs. (B) Corresponding box plot of R_u^2. Results correspond to the 40% FD threshold. Boxes annotated with an asterisk indicate a significant group difference (FDR p<0.05), while a circle indicates an initially significant difference between groups that did not survive FDR correction. Boxes show the quartiles of the dataset and whiskers show the distribution with the exception of outliers. Each dot is the mean R^2 from a single subject from all statistically significant grayordinates within each ROI.

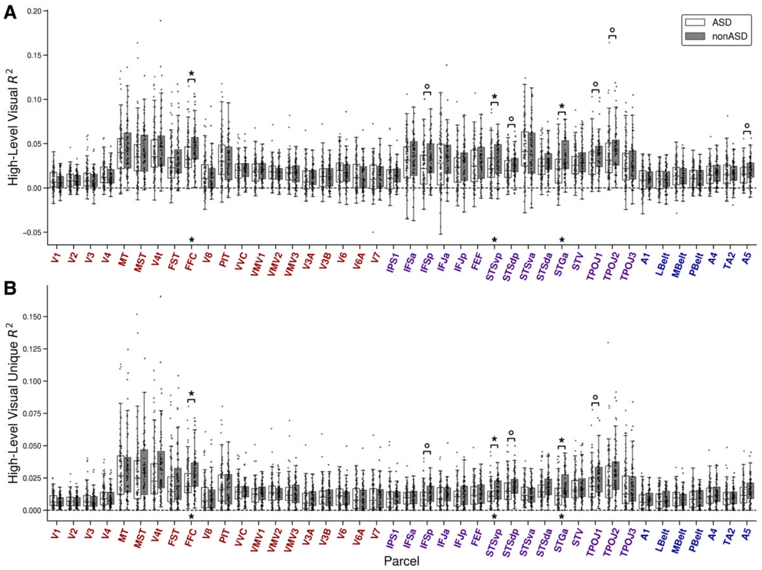

Figure 3—figure supplement 4. (A) Box plot of high-level visual encoding model R^2 for ASD (white) and nonASD (gray) groups across all perceptual ROIs. (B) Corresponding box plot of R_u^2. Results correspond to the 40% FD threshold. Boxes annotated with an asterisk indicate a significant group difference (FDR p<0.05), while a circle indicates an initially significant difference between groups that did not survive FDR correction. Boxes show the quartiles of the dataset and whiskers show the distribution with the exception of outliers. Each dot is the mean R^2 from a single subject from all statistically significant grayordinates within each ROI.

bioRxiv preprint doi: https://doi.org/10.64898/2026.03.23.713749; this version posted March 24, 2026. The copyright holder for this preprint (which was not certified by peer review) is the author/funder, who has granted bioRxiv a license to display the preprint in perpetuity. It is made available under aCC-BY 4.0 International license.

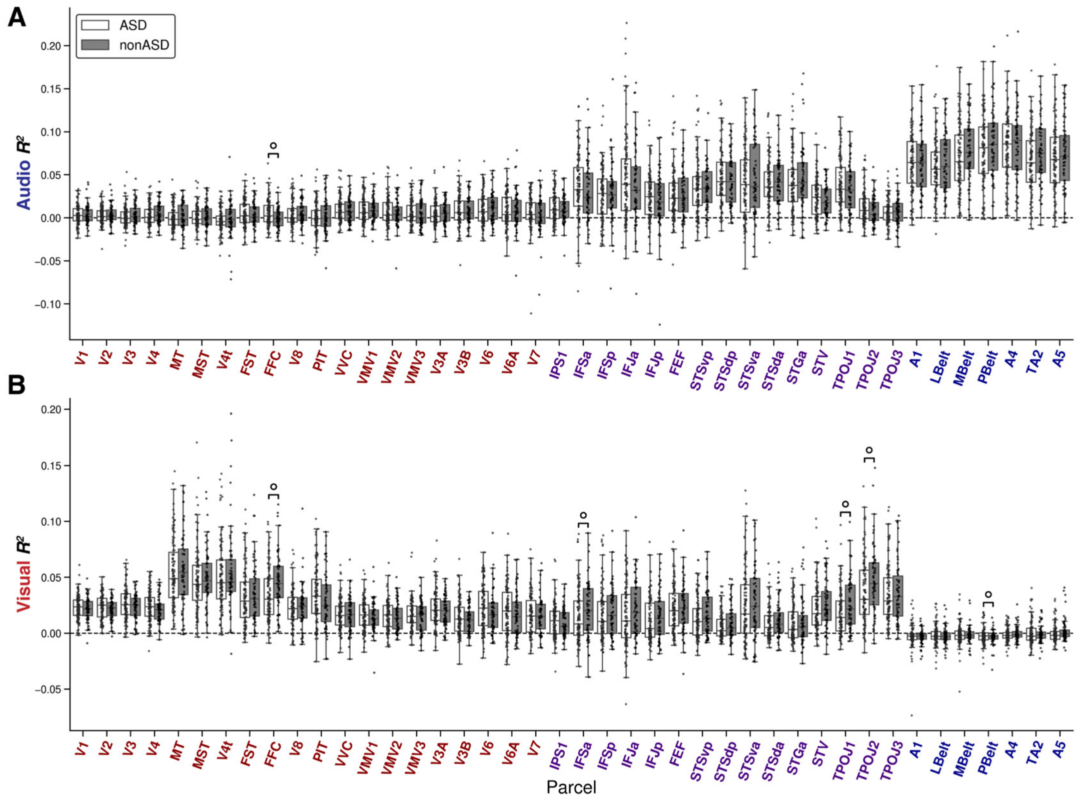

Figure 7—figure supplement 1. (A) Box plot of audio encoding model R^2 for ASD (white) and nonASD (gray) groups across all perceptual ROIs. (B) The same but for visual R^2. Results correspond with the 40% FD threshold. Boxes annotated with circles indicate a difference between groups that did not survive FDR correction (uncorrected p<0.05, FDR q>0.05). Boxes show the quartiles of the dataset and whiskers show the distribution with the exception of outliers. Each dot is the mean value from a single subject from all statistically significant grayordinates within each ROI.

bioRxiv preprint doi: https://doi.org/10.64898/2026.03.23.713749; this version posted March 24, 2026. The copyright holder for this preprint (which was not certified by peer review) is the author/funder, who has granted bioRxiv a license to display the preprint in perpetuity. It is made available under aCC-BY 4.0 International license.

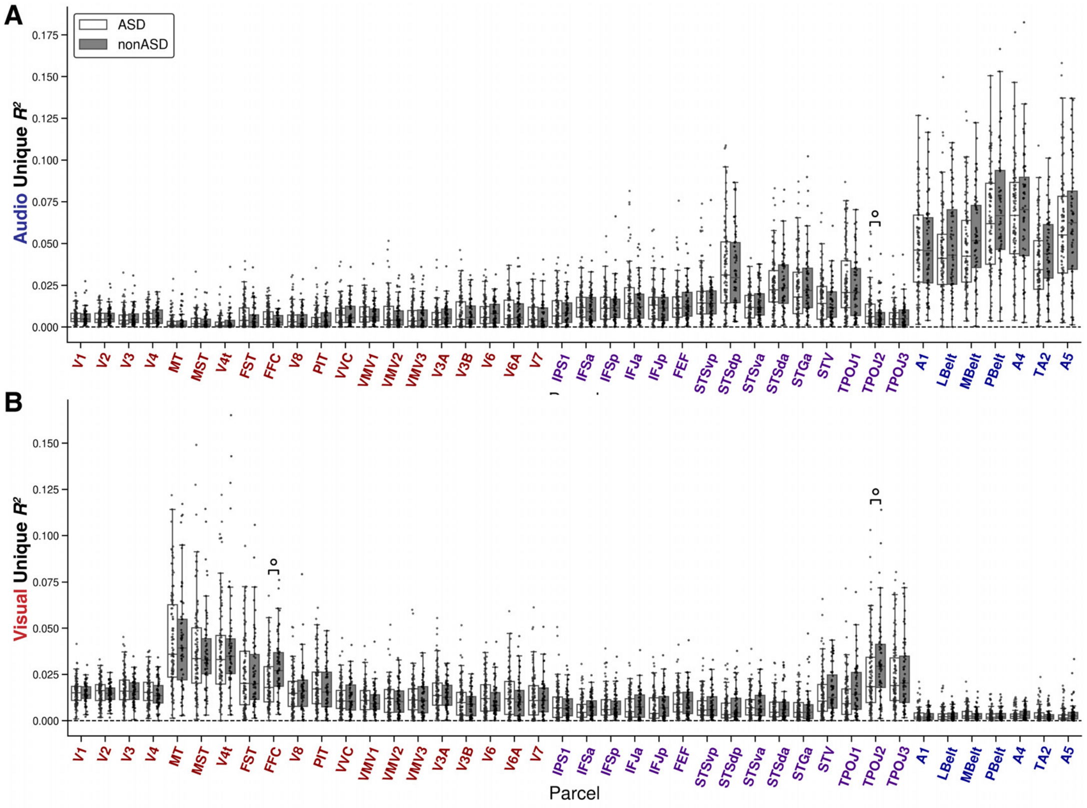

Figure 7—figure supplement 2. (A) Box plot of audio encoding model R_u^2 for ASD (white) and nonASD (gray) groups across all perceptual ROIs. (B) The same but for visual R_u^2. These results correspond with the 40% FD threshold. Boxes annotated with circles indicate a difference between groups that did not survive FDR correction (uncorrected p<0.05, FDR q>0.05). Boxes show the quartiles of the dataset and whiskers show the distribution with the exception of outliers. Each dot is the mean value from a single subject from all statistically significant grayordinates within each ROI.

bioRxiv preprint doi: https://doi.org/10.64898/2026.03.23.713749; this version posted March 24, 2026. The copyright holder for this preprint (which was not certified by peer review) is the author/funder, who has granted bioRxiv a license to display the preprint in perpetuity. It is made available under aCC-BY 4.0 International license.

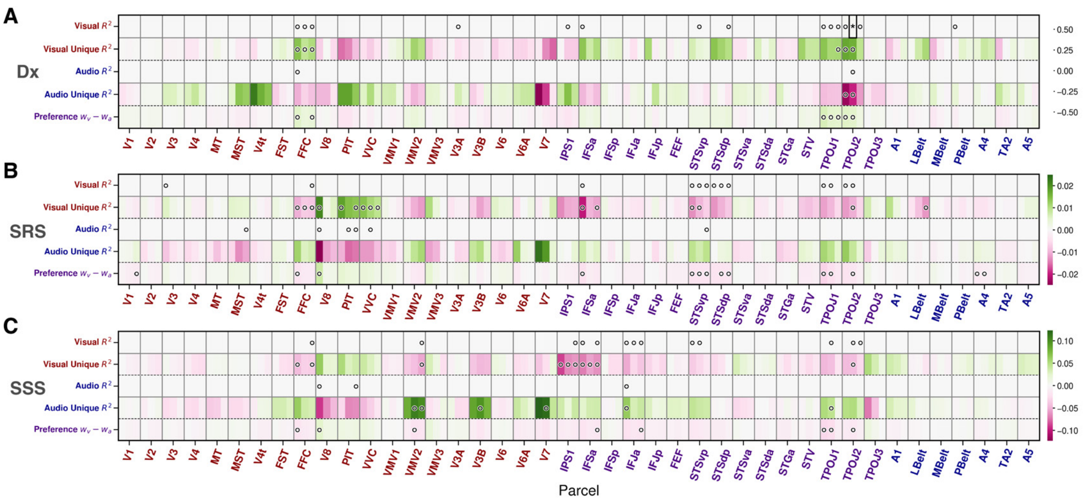

Figure 7—figure supplement 3. Heatmaps of fixed-effect coefficients for **(A)** diagnostic group (Dx: nonASD vs. ASD), **(B)** SRS Total T-Score and **(C)** sensory subset score (SSS), across 40%, 60% and 80% FD thresholds (left to right within each cortical parcel column). Within each of the three horizontal panels, rows denote encoding model-derived metrics (visual R^2 and R_u^2, audio R^2 and R_u^2, and their preference index ($W_V - W_A$) and columns denote Glasser MMP ROIs ordered left to right from early visual areas through association cortices followed by auditory areas. Color indicates the magnitude and sign of the coefficient (pink=negative effect with ASD>nonASD; green=positive effect with ASD<nonASD). Asterisks mark FDR-corrected significance at $q < 0.05$; open circles mark uncorrected $p < 0.05$. The visual modality encoding models are labeled in red and the auditory in blue.

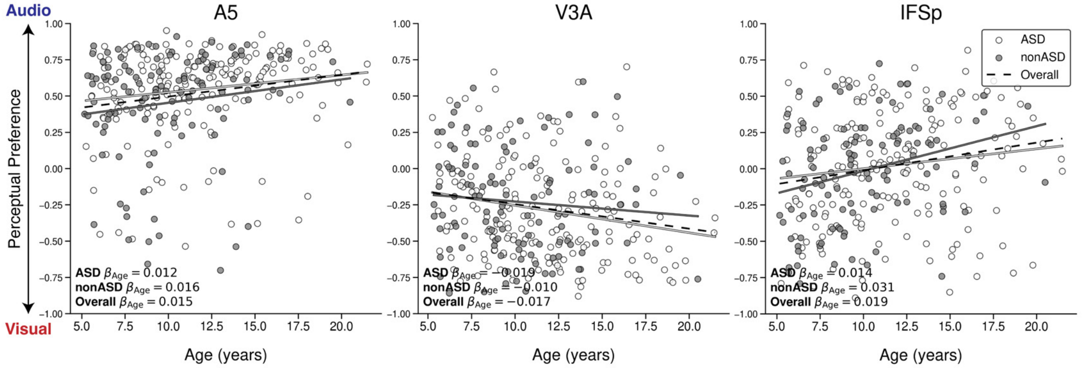

Figure 8—figure supplement 1. Scatter plots showing the relationship between age and perceptual preference in three example cortical regions (A5, V3A, and IFSp) where significant effects were observed across all participants. Perceptual preference values above zero indicate an auditory preference, and values below zero indicate a visual preference. Although no significant age-by-diagnosis interactions are displayed here, autistic (ASD; white) and non-autistic (nonASD; gray) groups are colored separately for clarity. Lines show model fits for each group colored correspondingly, with estimated age-related slopes (β_{Age}) reported for ASD, nonASD, and the overall sample (black dashed line).

bioRxiv preprint doi: https://doi.org/10.64898/2026.03.23.713749; this version posted March 24, 2026. The copyright holder for this preprint (which was not certified by peer review) is the author/funder, who has granted bioRxiv a license to display the preprint in perpetuity. It is made available under aCC-BY 4.0 International license.

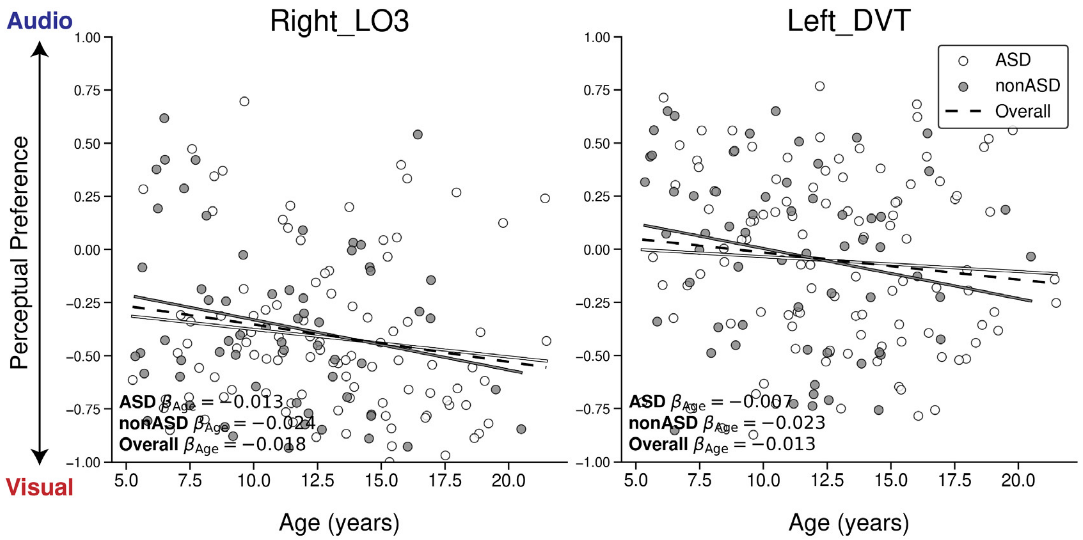

Figure 8—figure supplement 2. Scatter plots showing the relationship between age and perceptual preference in two example lateralized cortical regions, Right LO3 (a visual region between early visual cortex and MT+) and Left DVT (Dorsal Visual Transitional area; a region located on the posterior bank of the parieto-occipital sulcus), where significant effects were observed across all participants at the whole-brain level. Perceptual preference values above zero indicate an auditory preference, and values below zero indicate a visual preference. Although no significant age-by-diagnosis interactions are displayed here, autistic (ASD; white) and non-autistic (nonASD; gray) groups are colored separately for clarity. Lines show model fits for each group, with estimated age-related slopes (β_{Age}) reported for ASD, nonASD, and the overall sample (black dashed line).

bioRxiv preprint doi: https://doi.org/10.64898/2026.03.23.713749; this version posted March 24, 2026. The copyright holder for this preprint (which was not certified by peer review) is the author/funder, who has granted bioRxiv a license to display the preprint in perpetuity. It is made available under aCC-BY 4.0 International license.

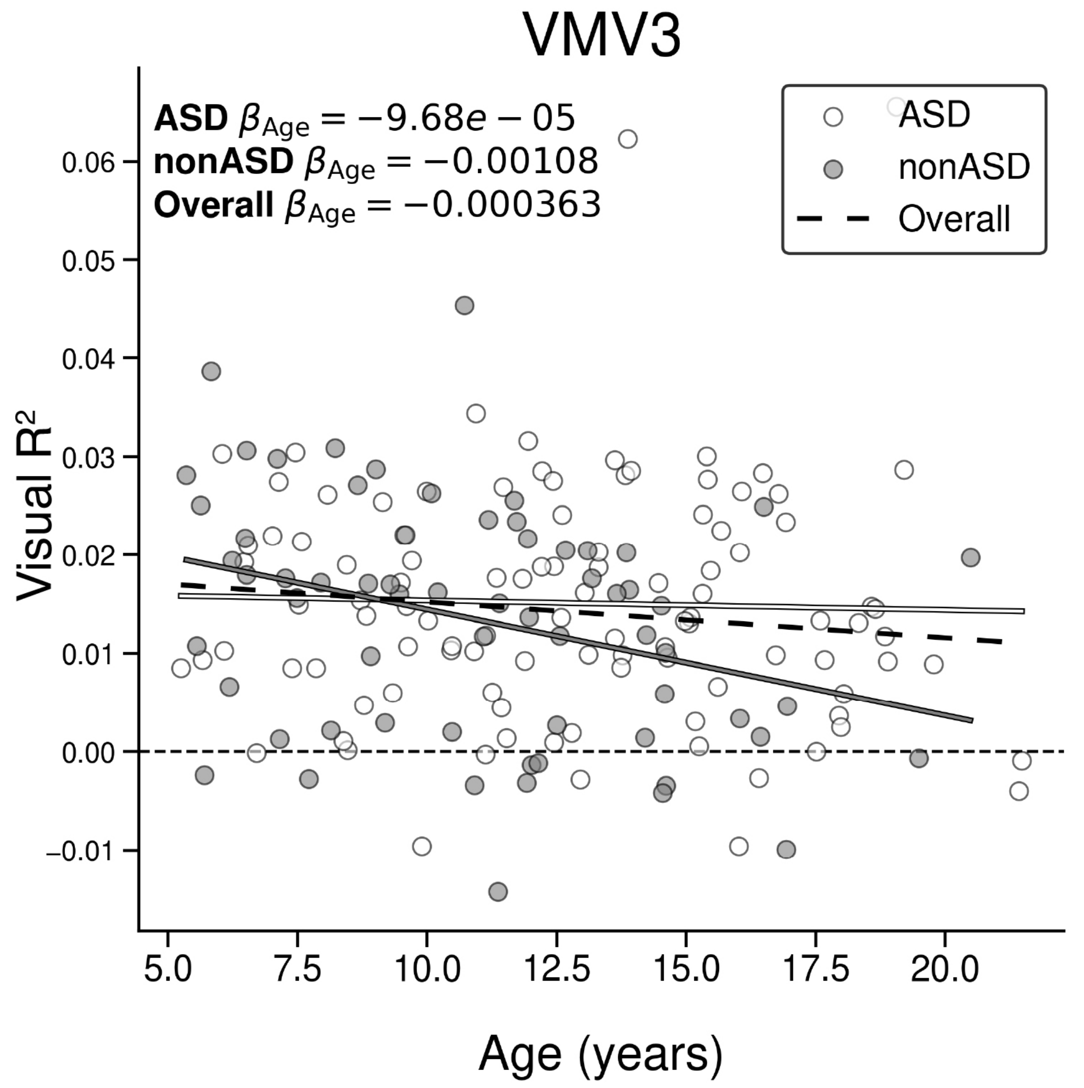

Figure 8—figure supplement 3. Scatter plot showing the relationship between age and visual R^2 in perceptual region VMV3 where a significant age:diagnosis interaction was observed. Autistic (ASD; white) and non-autistic (nonASD; gray) diagnostic groups are displayed. Lines show model fits for each group, with estimated age-related slopes (β_{Age}) reported for ASD, nonASD, and the overall sample (black dashed line).
